# Regular Proton-Pump Inhibitor Intake is Associated with Deterioration of Peripheral Bone Mineral Density, Microarchitecture, and Strength in Older Patients as Assessed by High-Resolution Peripheral Quantitative Computed Tomography (HR-pQCT)

**DOI:** 10.1007/s00223-025-01420-7

**Published:** 2025-10-22

**Authors:** Ursula Heilmeier, Alexandra Siegenthaler, Ursina Meyer, Marie Boensch, Melanie Kistler-Fischbacher, Caroline de Godoi Rezende Costa Molino, Bert van Rietbergen, Wei Lang, René Rizzoli, Robert Theiler, Andreas Egli, Heike Annette Bischoff-Ferrari

**Affiliations:** 1 Department of Geriatrics and Aging Research, University of Zurich, Zurich, Switzerland; 2 Centre on Aging and Mobility, University of Zurich and City Hospital Zurich, Zurich, Switzerland; 3 Musculoskeletal Quantitative Imaging Research Group, Department of Radiology & Biomedical Imaging, University of California San Francisco, San Francisco, USA; 4 Division of Internal Medicine, University Hospital Basel, Basel, Switzerland; 5 Department of Rheumatology and Clinical Immunology, Faculty of Medicine, Medical Center-University of Freiburg, University of Freiburg, Freiburg im Breisgau, Germany; 6 Department of Geriatrics, University of Basel, Basel, Switzerland; 7 University of Basel Department of Aging Medicine, FELIX Platter Basel, Basel, Switzerland; 8 Swiss Healthy Longevity Campus, University of Basel, Basel, Switzerland; 9 Center on Aging and Mobility, University of Zurich, Zurich, Switzerland; 10 Department of Biomedical Engineering, Eindhoven University of Technology, Eindhoven, The Netherlands; 11 Division of Bone Diseases, Geneva University Hospitals and Faculty of Medicine, Geneva, Switzerland

**Keywords:** Chronic PPI use, Bone microarchitecture, Bone biomechanics, Geriatrics, Vitamin D, High-resolution peripheral quantitative computed tomography, Adverse drug events, Randomized control trial

## Abstract

Proton-pump inhibitors (PPI) are widely used among older adults and PPI intake has been associated with increased fracture risk. However, their association with bone microarchitecture and strength remains unclear as prior clinical PPI studies were restricted to lower-resolution bone imaging techniques such as DXA. Using high-resolution peripheral computed tomography (HR-pQCT), we prospectively investigated whether long-term PPI use was associated with changes in volumetric bone mineral densities, bone microarchitecture, and strength in older patients. 189 patients aged ≥ 60 years (*n* = 24 persistent PPI users [p-PPI user], and *n* = 165 non-PPI users), enrolled as part of a 2-year vitamin D RCT, underwent HR-pQCT scanning of the tibia and radius at 0 and 2 years. Bone volumetric density (vBMD), microarchitectural and strength parameters were computed and adjusted linear regression models without and with adjustments, including age, sex, BMI, and treatment groups, were employed to compare changes between p-PPI users and non-PPI users. At baseline, both groups were comparable with respect to age, vBMDs, bone microarchitecture, and sex. During follow-up, p-PPI users lost significantly more distal tibial total volumetric BMD (ΔTt.BMD: − 8.58 vs. − 2.45 mg/cm^3^, *p* = 0.012) and cortical volumetric BMD (ΔCt.BMD, − 28.96 vs. -14.07 mg/cm^3^, *p* = 0.005), and showed a significantly greater loss in tibial bone strength (Δstiffness − 4848.1 vs. 20.7 N/mm, *p* = 0.029; Δfailure load − 204.5 vs. 16.7 N, *p* = 0.026) than non-PPI users. At the radius, p-PPI users showed over the 2 years significantly greater increases in cortical porosity and intracortical pore diameter than non-PPI users (ΔCt.Po, *p* = 0.005; ΔCt.PoDm, *p* = 0.009). Long-term PPI intake was associated with a significant decline in cortical microarchitecture at the radius and with a significant deterioration of volumetric bone mineral density and strength at the tibia in older patients.

**Supplementary Information:**

The online version contains supplementary material available at 10.1007/s00223-025-01420-7.

## Introduction

Osteoporosis is a common skeletal disease, which primarily affects older adults. According to estimates of the International Osteoporosis Foundation, one in two women and one in five men above the age of 50 years will experience an osteoporotic fracture throughout their residual lifespan [1]. Given the increased mortality and reduced quality of life associated with osteoporotic fractures [2,3], eliminating avoidable risk factors for osteoporosis is of utmost clinical priority.

Proton-pump inhibitors (PPI) are among the most frequently prescribed medications in older people and belong to one of the most prescribed medication globally [4]. In the US, 157 million prescriptions of PPIs were dispensed in 2012 [5], and the number of PPI prescriptions has since continued to rise in the US [6] and worldwide [7]. While PPIs are effective in treating acid-related disorders and in avoiding non-steroidal anti-inflammatory drug (NSAID)-related side effects, several lines of evidence have linked PPI usage to bone loss and to an increased risk for osteoporotic fractures. At a molecular level, in vitro studies suggest a direct deleterious inhibitory effect of PPIs on bone cells, such as osteoclasts and osteoblasts [8]. In humans, several recent observational studies reported a lower (areal) bone mineral density with PPI usage [9,10]. Furthermore, clinical cohort studies showed an association between PPI use and a higher risk of fragility fractures [11–13], particularly for long-term and high-dose PPI intake [14–17], while others failed to find such an association with regard to bone loss [18,19] and/or fractures risk [20,21]. These inconsistencies may be partly explained by the fact that those studies were either limited with respect to their PPI exposure time [18] or to lower-resolution bone imaging techniques, such as DXA or 3D quantitative computed tomography (3D-QCT) [19,22] and thus might not have been sensitive enough to characterize potential PPI-related bone microstructural and strength changes adequately.

The longitudinal association of PPIs intake and bone microarchitecture and bone strength has not been studied in humans. With the advent of high-resolution peripheral quantitative computed tomography (HR-pQCT) a powerful imaging modality is available that allows for cross-sectional and longitudinal in vivo quantification of bone microstructure and bone strength at an isotropic voxel size of 82 μm [23]. The aim of this study was to prospectively investigate over a 2-year period, whether a PPI intake of ≥ 50% of time during the 2-year study period was associated with changes in HR-pQCT-derived volumetric bone mineral density, bone microarchitecture, and strength in older patients.

## Materials and Methods

### Study Design and Participants

This study used data acquired in the Zurich Multiple Endpoint Vitamin D Trial (NCT00599807)—a two-year, double-blind, randomized-controlled trial (RCT) aimed primarily at investigating the effect of two different daily doses of Vitamin D (a standard daily vitamin D dose of 800 IU and a high daily vitamin D dose of 2000 IU) on knee pain and functional performance in 273 patients, ≥ 60 years, who had undergone unilateral total knee replacement due to severe osteoarthritis [24]. All participants received additionally 500 mg of supplemental calcium (calcium carbonate, Sandoz) per day. The study took place between 2008 and 2014 at two hospital centers in Zurich. A detailed description of the screening procedure and of the eligibility criteria was published earlier [24]. In brief, patients were eligible if they were men or women aged 60 years or more and scheduled for unilateral total knee replacement due to severe knee OA and had no plans for bilateral knee replacement within the next 2 years. Additionally, patients had to be cognitively healthy (as shown by at least 24 out of 30 points on the Mini-Mental State Examination), mobile, and willing to halt current vitamin D and calcium supplementation during the trial. Key bone-related exclusion criteria comprised the presence of chronic kidney disease stage 4 or higher (creatinine clearance < 30 ml/min/1.73 m^2^), the presence of inflammatory arthritis, malabsorption disorders, hypercalcemia (serum albumin-adjusted calcium > 2.8 mmol/l), cancer, a weekly alcohol consumption of > 21 drink-units, or a medication use with known impact on bone metabolism, such as a chronic corticosteroid use, treatment with oral bisphosphonates, PTH or calcitonin in the 6 months prior to enrollment, or a zoledronate injection within the year prior to enrollment [24].

### Study Visits and Assessment of Baseline Variables and Covariates

All patients were seen for their initial clinical baseline visit, which took place 6–10 weeks after their total knee arthroplasty surgery and during which age, sex and smoking status, and the number of comorbidities using the Charlson comorbidity index [25] were assessed by questionnaire. Over the 2-year follow-up period, patients were then evaluated every 6 months in a separate clinical study visit which included a blood draw, a physical examination and the administration of questionnaires. In addition, participants were contacted by study nurses every 2 months via phone to assess the medication usage of the past two months, incident falls, adverse events (including fractures), and adherence to the study medication. The well-validated Western Ontario and Mc Master Universities (WOMAC) Osteoarthritis pain and functional domain scores [26] were assessed at the baseline visit (6–10 weeks postknee arthroplasty) separately for the operated knee and the non-operated knee via a self-administered questionnaire. For the WOMAC pain domain score, 5 questions referring to pain levels during the past 4 weeks prior to the visit were asked. These encompassed the questions if the participant had experienced during the course of the past 4 weeks: (1) any pain during walking, (2) any pain using stairs, (3) any pain in bed during the night, (4) any pain while sitting or lying down, and (5) any pain while standing upright. Each question was rated on a scale from 0 to 4 (none [0 point]), mild [1 point], moderate [2 points], severe [3 points], and extreme pain [4 points]), and the final WOMAC pain domain score (score 0–100) was computed by summing the individual scores for each of the 5 questions and multiplying them by 5. For the WOMAC functional domain score, a total of 17 questions was assessed regarding how difficult it had been over the past 4 weeks to, e.g., use stairs, rise from sitting, to stand, to bend to the floor, to walk on an flat/plan ground, to get in/out of a car or bus, to do shopping, to put on/take off socks, to rise from bed, to lie in bed, to get in/out of the bath, to sit for a long time, to get on/off the toilet, to carry out heavy domestic duties, and to carry out Light domestic duties. Each question was again rated on a scale from 0 to 4 [not difficult (0 pt), mildly difficult (1 pt), moderately difficult (2 pts), severely difficult (3 pts), and extremely difficult (4 pts)], and the final WOMAC functional domain score was computed by adding the individual scores for each of the 17 questions and multiplying them by 1.47. The WOMAC scores therefore range from 0 to 100 for each domain. Bodyweight (kg) and height (cm) were measured at baseline and at follow-up and the body mass index (BMI) was calculated accordingly (kg/m^2^).

### Assessment of Exposure: Proton-Pump Inhibitor Use

At baseline, every 2 months via the phone calls, and at the 6-, 12-, 18-, and 24-month follow-up visits, self-reported intake of prescription-medications and of over-the-counter medications, including herbal medicines, homeopathic, and nutritional supplements, were recorded. For each medication, the brand name, generic name, the Anatomical Therapeutic Chemical Classification (ATC) code, as well as the indication were recorded. For the present analysis of proton-pump inhibitor usage, we only allowed medications with an ATC code of A02BC01-11 (excluding A02BC08) and combined NSAID/M01AE52, M01AE56, corresponding to the pharmacological class of proton-pump inhibitors. For NSAID medications, we considered all medications with an ATC code of M01A. As the association of PPIs on bone microarchitecture has not been studied yet longitudinally and as previous DXA-based studies had suggested a strong positive association between PPI dose, length of intake, and fracture risk, we wanted to focus in our study only on examining the most extreme ends of our study population spectrum, namely those with more than 50% days of PPI usage during the 2-year study period and those without PPI usage. P(ersistent)-PPI users (p-PPI user) had to have any persistent PPI usage on > 50% days over the entire 2-year study period. This could also include PPI usage at baseline. To be eligible for the non-PPI user group, study participants had to be free of any PPI intake at baseline and over the 2-year study period. Data from participants with occasional PPI use (> 0 to < 50% days of study time) were excluded from the present analysis.

### Assessment of Outcomes: Laboratory Parameters

At each clinical visit, fasting venous blood was drawn and further processed into serum. Serum samples were shipped immediately to the Institute of Clinical Chemistry at the University Hospital Zurich, where levels of serum (albumin-adjusted) calcium, phosphorus, bone-specific alkaline phosphatase (BAP), and intact parathyroid hormone (PTH) were quantified on a Cobas 8000 analyzer using assays from Roche Diagnostics (Rotkreuz, Switzerland). Estimated glomerular filtration rate (eGFR) was calculated using the CKD-EPI-eq. [27]. As the methodology for BAP analysis changed over the 2-year study period, the Passing–Bablok equation (*y* = 1.14x – 1.11 (*r* = 0.934)) was employed to correct baseline measurements. To quantify serum 25(OH) vitamin D concentrations, samples were first stored at − 80 °C and then analyzed as a batch after the trial via a sensitive and selective ultra-HPLC–MS/MS system [28].

### Assessment of Outcomes: Areal and Volumetric Bone Mineral Density, Bone Microarchitecture, and Bone Strength

#### DXA Measurements

At baseline, areal bone mineral density (aBMD, g/cm^2^) was measured in each patient at the lumbar spine and hip via the same dual-energy X-ray absorptiometry (DXA) system (Hologic 4500 Discovery Version 12.1; Hologic, Inc., Bedford, MA, USA). DXA-outcome parameters encompassed the areal BMD at the lumbar spine, femoral neck, and total hip. The lowest baseline T-score of these three sides was used to classify participants as osteoporotic (T-score ≤ − 2.5), osteopenic (T-score between − 1 and − 2.5), and normal (T-score ≥ − 1).

#### HR-pQCT Imaging

At baseline and at the 2-year follow-up, patients underwent high-resolution peripheral quantitative computed tomography (HR-pQCT) scanning at the standard distal tibia and radius scan sites. All scans were performed using the same clinical first-generation high-resolution peripheral quantitative computed tomography system (HR-pQCT I) manufactured by Scanco Medical AG (XtremeCT I, Scanco Medical AG, Brüttisellen, Switzerland) as previously described [29]. The tibia of the non-operated lower limb and the radius of the non-dominant upper limb were scanned. To calculate densitometric bone parameters, image attenuation values were calibrated against the attenuation values derived from a standardized hydroxyapatite (HA) phantom that was imaged daily on the scanner for quality control [30].

#### Image Postprocessing Including Standard and Micro-finite-element (µFE) Analysis

Details of image postprocessing including information on image segmentation, image registration, as well as standard bone microstructural and extended analysis are provided in the supplemental material. In brief, images were contoured semi-automatically after a visual image quality check and the manufacturer’s standard 2D registration technique was applied to match baseline and follow-up images. All HR-pQCT images were then further analyzed using the same, well-established standard image evaluation protocol [31,32] and standard bone parameters such as the total volumetric bone mineral density (vBMD), cortical vBMD (Ct.vBMD), and cortical area (Ct.Ar), cortical porosity (Ct.Po) and cortical pore diameter (Ct.Po.DM) as well as trabecular parameters (trabecular number [Tb.N], trabecular thickness [Tb.Th], and trabecular spacing [Tb.Sp]) were computed using the methods described by Burghardt and colleagues [33].

For µFE analysis, linear micro-finite-element analysis (µFEA) modeling was performed as detailed previously [33] and the biomechanical indices stiffness *K* and the failure load *F* were calculated for each scan site. Intrascanner reproducibility of HR-pQCT measurements is 0.8–2.0% RMS-CV% for density parameters, < 5% RMS-CV% for microstructural parameters, and 2.0–3.5% RMS-CV% for biomechanical parameters, respectively [34].

#### Statistical Analysis

Normal distribution of parameters was checked visually via histograms and Q–Q plots, and statistically via Shapiro–Wilk tests. Between-group differences in baseline characteristics, such as demographics, anthropometrics, health status, bone parameters, and baseline laboratory values, were assessed using a χ^2^ test for categorical variables and independent Student’s t tests for continuous variables. We employed Linear regression models to address the differences in outcome changes from baseline to 2 years between p-PPI users and non-PPI users. To additionally assess robustness of our skeletal findings, three different linear regression models were run: model 0 (without any adjustments), model 1 (with adjustments for age, BMI and sex), and model 2 with adjustments for age, sex, BMI, vitamin D treatment group, number of comorbidities, and baseline levels of the respective bone parameter. Changes in serum parameters between both groups were computed using Linear regression models adjusted for age, sex, BMI, vitamin D treatment group, number of comorbidities, and baseline levels of the respective serum outcome parameter. Changes in outcome measures across the 2 years are presented as least square means.

To correct for multiple comparisons while taking into account the hierarchical specifics of bone tissue and of HR-pQCT-derived bone parameters, we applied the hierarchical false discovery rate (FDR) controlling method as published by Yekutieli [35] and as suggested in the latest HR-pQCT guidelines [36] as a valuable option to account for multiplicity of comparisons in HR-pQCT data. Applying a standard false discovery rate of 0.05, and similar to the HR-pQCT study by Gensburger et al. [37], we tested our data in 3 levels. At the first level, four parameters were tested simultaneously: Δ total vBMD at the tibia, Δ total vBMD at the radius, Δ stiffness at the tibia and Δ stiffness at the radius. At the second level, Δ cortical vBMD at the tibia and at the radius, Δ trabecular vBMD at the tibia and at the radius, as well as Δ estimated failure load at the tibia and at the radius were tested only if the site-specific differences of Δ total vBMD had been statistically significant. We next tested on the third level for changes in trabecular bone microstructural parameters (Δ Tb.Th, Δ Tb.N, Δ Tb.Sp) only if the site-specific Δ trabecular vBMD had been statistically significant and for changes in cortical microstructural parameters (Δ Ct.Th, Δ Ct.Po, Δ Ct.Po.Dm) only if the site-specific change in cortical vBMD had been statistically significant. Statistical analysis was performed using the SAS v 9.4 (SAS Institute, Inc, Cary, NC) software package and statistical significance was set at *P* value of ≤ 0.05. All reported *P* values are two-sided.

## Results

### Participant Characteristics

**Fig. 1:**
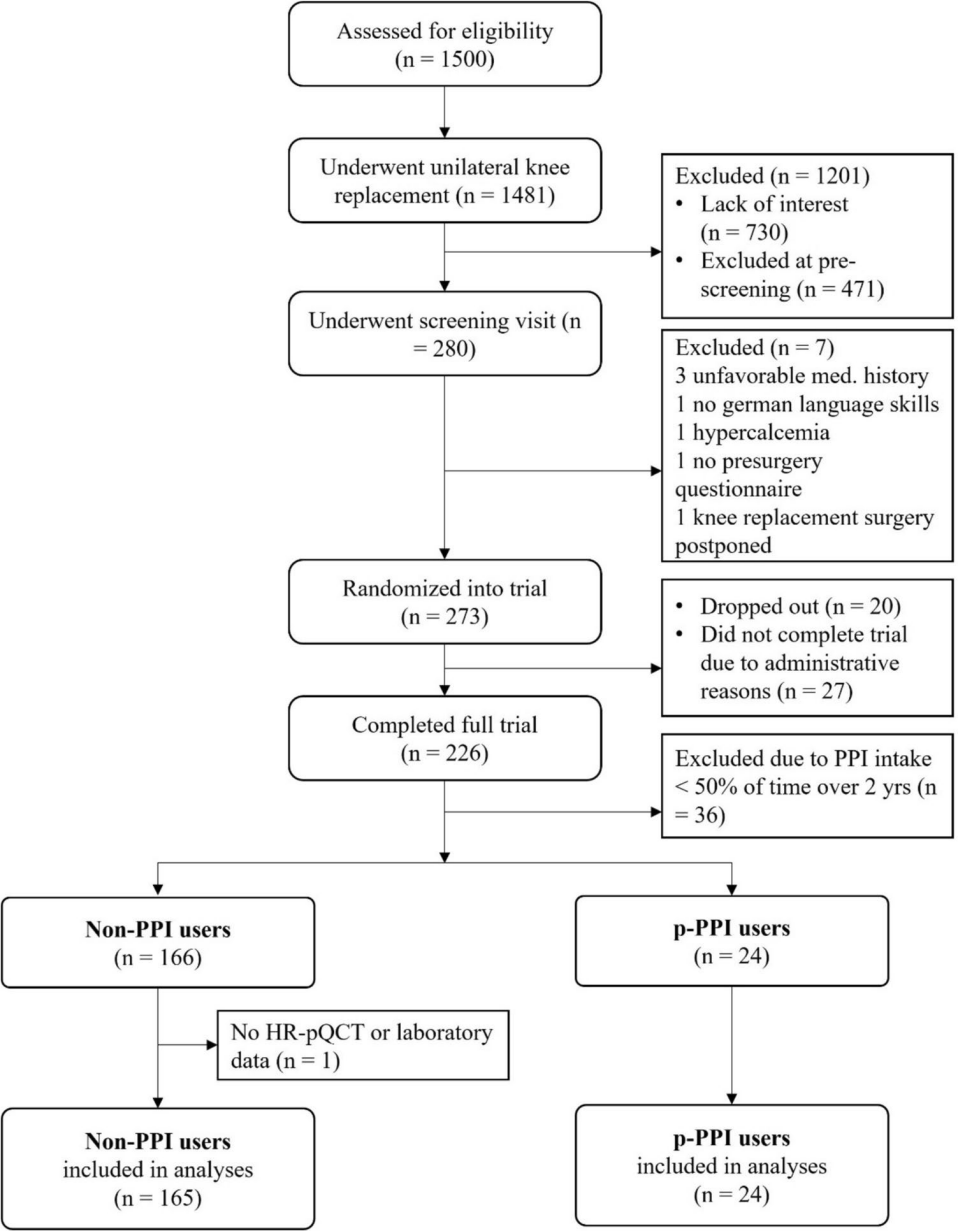
Flowchart illustrating participant selection. Detailed information on eligibility criteria and reasons for dropouts/participant exclusion have been published previously [24]. *HR-pQCT* high-resolution peripheral quantitative computed tomography, *non-PPI users* non-users of proton-pump inhibitors, *p-PPI users* persistent proton-pump inhibitor users

A flowchart of the detailed patient selection is given in Fig. 1. Of the 273 participants recruited and randomized into either the Vit D 2000 IU or the Vit D 800 IU daily group of the trial, a total of 226 participants completed the full 2-year course of the trial. 47 patients did not finish the trial protocol as they either dropped out (*n* = 20) over the 2-year trial period either due to unwillingness to continue the study (*n* = 12), death (*n* = 4), other health reasons (*n* = 3), or loss for follow-up (*n* = 1), or administrative reasons (*n* = 27) [24]. Based on their PPI usage during the 2-year study period, the 226 participants were then stratified either into the non-PPI user group (*n* = 166 patients who did not take any PPI at all during the 2-year study period), and the persistent PPI-user group (p-PPI users), who took PPIs for more than 50% (50 or more) of the days during the 2-year study period. We excluded 36 participants who reported occasional PPI use (< 50% of the study time), leaving a total of 166 non-PPI users and a total of 24 p-PPI users for further HR-pQCT and laboratory analysis. One patient of the non-PPI user group was not included into HR-pQCT image analysis due to a prior distal tibial fracture at the scan site. This resulted in a final total sample of 189 patients (*n* = 165 non-PPI users, and *n* = 24 p-PPI users) who completed the study and had HR-pQCT scans at baseline and at the 2-year follow-up available.

Baseline clinical and laboratory characteristics of the 189 study participants are given in Table 1. Overall, participants’ mean age was 70.8 ± 6.6 years and they were overweight with BMIs ranging between 27.3 ± 4.0 kg/m^2^. 51.9% of all study participants were women and 48.1% were men. 51.9% of all study participants received treatment with 800 IU vitamin D3 daily, and the residual 48.1% were treated with a daily supplement of 2000 IU vitamin D3. Persistent-PPI users and non-PPI users exhibited similar characteristics at baseline in terms of demographics, anthropometrics, and general health: both groups displayed a similar baseline mean age (71.4 years vs. 70.7 years in p-PPI group versus non-PPI users, *p* = 0.644) and height and were in good overall health, with most participants in each group being free of any comorbidity as Listed in the Charlson comorbidity index. Of the non-PPI users, none took PPIs at baseline, while of the PPI users 17 (70.8%) were taking PPI at baseline. The proportion of NSAID users was significantly higher in the p-PPI group, than in the non-PPI group at baseline (*p* = 0.041) and at 2-year follow-up (*p* < 0.001).

**Table 1:** Baseline characteristics of all study participants (*n* = 189), overall and by PPI use

	Overall (*n* = 189)	Non-PPI users (*n* = 165)	p-PPI users (*n* = 24)	*p* values ɫ
Demographics and anthropometrics				
Age [years]	70.8 ± 6.6	70.7 ± 6.7	71.4 ± 6.1	0.644
Height [cm]	169 ± 9.0	168 ± 9.0	170 ± 8.0	0.431
Weight [kg]	77.8 ± 14.1	76.8 ± 13.7	84.9 ± 14.6	**0.008**
Δ Weight over 24 months [kg]	1.5 ± 3.7	1.6 ± 3.6	1.3 ± 4.4	0.707
Body mass index [kg/m^2^]	27.3 ± 4.0	27.0 ± 3.9	29.3 ± 4.0	**0.008**
Δ BMI over 24 months [kg/m^2^]	0.6 ± 1.3	0.6 ± 1.3	0.6 ± 1.6	0.891
Sex: Female n [%]	98 (51.9)	86 (52.1)	12 (50)	0.846
Male n [%]	91 (48.1)	79 (47.9)	12 (50)	
Treatment arm allocation				0.846
Vit D3 2000 IU/daily [%]	48.1	47.9	50	
Vit D3 800 IU/daily [%]	51.9	52.1	50	
Health status as assessed via Charlson comorbidity index (score 0–37)				
Average number of comorbidities	0.6 ± 1.0	0.6 ± 1.0	0.8 ± 1.1	0.378
Percentage of patients with n comorbidities				0.935
0	65.1 (%)	65.5 (%)	62.5 (%)	
1–2	31.7 (%)	31.5 (%)	33.3 (%)	
3 +	3.2 (%)	3.0 (%)	4.2 (%)	
WOMAC scores (score 0–100)				
Pain operated knee at baseline visit	28.5 ± 15.0	28.7 ± 15.1	26.9 ± 14.0	0.573
Pain non-operated knee at baseline visit	4.6 ± 8.3	4.8 ± 8.4	3.3 ± 7.0	0.426
Function operated knee at baseline visit	25.3 ± 13.7	25.2 ± 13.6	26.2 ± 14.2	0.736
Function non-operated knee at baseline visit	4.0 ± 8.2	4.0 ± 8.2	3.6 ± 8.1	0.830
Medication intake at baseline				
NSAID intake at baseline, n [%]	22 (11.6)	16 (9.7)	6 (25.0)	**0.041**
NSAID intake at 2 years, n [%]	19 (10.1)	11 (6.7)	8 (33.3)	** < 0.001**
Bone health status based on				
Baseline DXA (lowest) T-score				
Normal T-score ≥ -1.0 [%]	42.9	41.2	54.2	0.456
Osteopenic T-score < -1.0, > -2.5 [%]	49.7	50.9	41.7	
Osteoporotic T-score ≤ -2.5 [%]	7.4	7.9	4.2	
Femur neck T-score	− 1.0 ± 1.0	− 1.1 ± 0.9	− 0.7 ± 1.1	*0.068*
Total hip T-score	− 0.4 ± 1.1	− 0.5 ± 1.0	0.0 ± 1.1	**0.032**
Lumbar spine T-score	0.1 ± 1.7	0.1 ± 1.7	0.2 ± 1.6	0.777
Serum laboratory measurements				
Calcium [mmol/l]	2.33 ± 0.09	2.33 ± 0.09	2.33 ± 0.11	0.803
Calcium (24 months) [mmol/l]	2.34 ± 0.10	2.33 ± 0.10	2.37 ± 0.11	*0.053*
PTH intact, [ng/l]	49.0 ± 18.2	48.3 ± 18.1	54.2 ± 17.9	0.136
25-OH Vitamin D3 [ng/ml]	27.1 ± 12.2	27.8 ± 12.4	22.7 ± 10.2	*0.056*
Phosphorus [mmol/l]	1.0 ± 0.2	1.0 ± 0.1	1.0 ± 0.1	0.504
Bone-specific alkaline phosphatase [μg/l]	13.7 ± 6.1	13.5 ± 5.0	15.4 ± 11.2	0.432
eGFR [ml/min/1.73 m^2^]	80.52 ± 14.8	81.11 ± 14.5	76.52 ± 16.2	0.157
eGFR (24 months) [ml/min/1.73 m^2^]	78.87 ± 15.4	79.80 ± 14.9	72.52 ± 17.8	**0.030**

Data are expressed as mean ± SD unless otherwise stated

**ɫ** p-PPI users vs. non-PPI users. Results in bold font indicate statistically significant differences between p-PPI users and non-PPI users

Δ delta change in parameter, *BMI* body mass index, *DXA* dual-energy X-ray absorptiometry, *eGFR* estimated glomerular filtration rate, *non-PPI users* non-users of proton-pump inhibitors, *NSAID* non-steroidal anti-inflammatory drug, *p-PPI users* persistent PPI users, *PTH* parathyroid hormone, *WOMAC* Western Ontario and McMaster Universities Arthritis Index

For the operated knee, both groups reported at baseline comparable WOMAC pain score levels that ranged between 26.9 ± 14.0 (p-PPI group) and 28.7 ± 15.1 out of 100 (non-PPI group, *p* = 0.573) (Table 1). For the non-operated knee (and therefore at the HR-pQCT measured leg), WOMAC pain levels at baseline were similarly low in both groups ranging between 3.3 ± 7.0 out of 100 for the p-PPI group and 4.8 ± 8.4 out of 100 for the non-PPI group, respectively (*p* = 0.736). With respect to lower extremity function as assessed via the WOMAC functional domain score, we observed that both groups exhibited at the operated knee at baseline a similar magnitude of functional impairment, while functional impairment at the non-operated (HR-pQCT measured) leg was and remained minimal and comparable in both groups (p-PPI-group: 3.6 ± 8.1 vs. non-PPI group: 4.0 ± 8.2. out of 100 in WOMAC function score, *p* = 0.830, Supplemental Table A). With respect to sex and treatment arm assignment, both groups showed comparable proportions of participants in each treatment arm and a similar sex distribution (50% females in the p-PPI group vs. 52.1% in the non-PPI group, *p* = 0.846). Regarding bone health and metabolism, mean baseline 25(OH) vitamin D serum levels of both p-PPI users and non-PPI users were in the vitamin D insufficient range (below 30 ng/mL). Apart from that, both groups were normocalcemic, and had normal ranging baseline levels of serum phosphorus, intact PTH, and bone-specific alkaline phosphatase (BAP), as well as comparable eGFR levels (all *p* > 0.05) at baseline. The main anthropometric difference between groups manifested in a significantly higher baseline weight and BMI in the p-PPI group relative to non-PPI users. However, both groups showed a comparable amount of weight gain and BMI gain during the 2-year follow-up time (Table 2, *p* > 0.05).

**Table 2:** Baseline volumetric bone mineral density, bone microarchitectural and bone strength parameters as measured via HR-pQCT at the distal tibia and radius of all study participants (n = 189), overall and by PPI use

Distal tibia	Overall (*n* = 189)	Non-PPI users (*n* = 165)	p-PPI users (*n* = 24)	*p *values ɫ
Basic HR-pQCT measures				
Tt.BMD [mg/cm^3^]	278.0 ± 56.7	278.9 ± 57.8	270.8 ± 48.5	0.546
Tb.BMD [mg/cm^3^]	168.9 ± 37.5	168.4 ± 37.7	172.4 ± 36.6	0.658
Ct.BMD [mg/cm^3^]	827.7 ± 68.1	830.8 ± 67.4	804.5 ± 70.0	0.105
Ct.Th [mm]	1.2 ± 0.3	1.2 ± 0.3	1.2 ± 0.3	0.580
Ct.Po [%]	10.2 ± 4.4	10.0 ± 4.4	11.3 ± 3.8	0.239
Ct.Po.Dm [µm]	204.8 ± 28.3	204.7 ± 28.6	205.9 ± 26.3	0.855
Tb.N [mm-1]	1.9 ± 0.4	1.9 ± 0.3	1.9 ± 0.4	0.480
Tb.Th [µm]	75.2 ± 12.2	75.3 ± 12.3	74.80 ± 11.6	0.864
Tb.Sp [µm]	477.7 ± 118.8	478.5 ± 114.7	471.1 ± 149.4	0.794
Biomechanics				
Stiffness, K [kN/mm]	219.4 ± 56.6	218.5 ± 56.4	226.3 ± 59.4	0.563
Estimated failure load, F [kN]	11.1 ± 2.8	11.0 ± 2.8	11.4 ± 2.9	0.556
Distal radius				
Basic HR-pQCT measures				
Tt.BMD [mg/cm^3^]	318.6 ± 76.6	320.9 ± 78.4	301.3 ± 60.9	0.285
Tb.BMD [mg/cm^3^]	158.7 ± 46.6	158.7 ± 47.7	158.7 ± 38.6	0.995
Ct.BMD [mg/cm^3^]	876.8 ± 70.9	880.1 ± 71.3	852.5 ± 64.9	0.102
Ct.Th [mm]	0.9 ± 0.2	1.0 ± 0.2	0.9 ± 0.2	0.455
Ct.Po [%]	4.1 ± 2.2	4.0 ± 2.1	4.7 ± 2.3	0.581
Ct.Po.Dm [µm]	188.2 ± 35.8	188.2 ± 37.0	188.5 ± 26.7	0.967
Tb.N [mm-1]	1.9 ± 0.4	1.9 ± 0.4	2.0 ± 0.3	0.341
Tb.Th [µm]	68.5 ± 13.9	68.8 ± 14.2	66.4 ± 12.0	0.468
Tb.Sp [µm]	489.2 ± 200.7	494.2 ± 210.3	451.8 ± 100.4	0.376
Biomechanics				
Stiffness, K [kN/mm]	88.4 ± 25.7	88.4 ± 25.9	89.2 ± 24.7	0.895
Estimated failure load, F [kN]	4.5 ± 1.3	4.5 ± 1.3	4.5 ± 1.2	0.819

Data are expressed as mean ± SD

ɫ persistent PPI users vs. non-PPI users

*Ct.BMD* volumetric BMD of the cortical compartment, *Ct.Po* intracortical porosity, *Ct.PoDm* mean cortical pore diameter, *Ct.Th* cortical thickness, *HR-pQCT* high-resolution peripheral quantitative computed tomography, *non-PPI users* non-users of proton-pump inhibitors, *p-PPI users* persistent PPI users, *Tb.BMD* volumetric BMD of the trabecular compartment, *Tb.N* trabecular number, *Tb.Sp* trabecular separation, *Tb.Th* trabecular thickness, *Tt.BMD* total volumetric bone mineral density including trabecular and cortical bone

### Baseline Bone Health Assessed by Dual-Energy X-Ray Absorptiometry (DXA)

Based on the lowest T-score of all 3 baseline DXA measurement sites, we found that p-PPI and non-PPI groups showed comparable proportions of osteoporotic, osteopenic, and healthy participants (4%, 42%, and 54% versus 8%, 51%, and 41%, *p* = 0.456). When looking at the different DXA sites, p-PPI users exhibited slightly higher T-scores at the femoral neck and total hip relative to non-PPI users, reaching statistical significance at the total hip (*p* = 0.032). The baseline T-score of the lumbar spine was comparable between both groups.

### Baseline Skeletal Characteristics as Assessed by HR-pQCT

#### Baseline Density, Cortical and Trabecular Microstructural Parameters

When examining baseline volumetric bone mineral density, cortical and trabecular parameters (Table 2), we observed that at both scan sites total volumetric BMD, cortical, and trabecular volumetric BMD were comparable between p-PPI users and non-PPI users. Similarly, no differences were observed for baseline cortical (Ct.Th., and Ct.Po.) and trabecular microstructural parameters (Tb.Th., Tb.N., Tb.Sp.).

#### Baseline Bone Strength Parameters

At baseline, there were no significant differences in biomechanical HR-pQCT parameters, such as stiffness and failure load between the two groups at either of the two scan sites (Table 2).

#### Longitudinal Changes in Serum Bone Metabolic Markers

Adjusted least square means of changes in serum bone metabolic markers are shown in Table 3. Over the 2-year study period, we detected a slight, but significant increase in serum calcium levels (*p* = 0.040) and a small, but significant reduction in eGFR levels (*p* = 0.035) in the p-PPI group compared to non-PPI users. No other significant differences in laboratory changes were noted during the follow-up for any other bone metabolic markers such as intact PTH, BAP or serum phosphorus levels among groups. This was also the case for changes in 25(OH) vitamin D_3_ serum levels: with an overall study medication adherence rate of 93% [24] and similar proportions of participants receiving either 800 IU or 2000 IU of vitamin D daily in both PPI groups, increases in serum 25(OH) vitamin D3 levels over the 2-year study period were similar in the 2 groups.

**Table 3:** Adjusted least square means of changes (Δ) in serum bone metabolic markers over the 2-year follow-up period of all study participants (*n* = 189), by PPI use

	Adjusted mean absolute change (Δ) [95% CI]		
	Non-PPI users (*n* = 165)	p-PPI users (*n* = 24)	*p* value ɫ
Serum laboratory markers			
Δ Calcium (mmol/l)	0.00 [− 0.01, 0.01]	0.04 [0.00, 0.07]	**0.040**
Δ PTH intact, (ng/l)	1.14 [− 0.72, 3.0]	4.22 [− 0.70, 9.13]	0.251
Δ 25-OH Vitamin D3 (ng/ml)	14.59 [13.3, 15.9]	12.56 [9.1, 16.0]	0.282
Δ Phosphorus (µmol/l)	− 9.61 [− 27.8, 8.6]	− 18.95 [− 64.7, 26.8]	0.710
Δ Bone-specific alkaline phosphatase (μg/l)	− 3.11 [− 3.6, − 2.6]	− 2.50 [− 3.9, − 1.1]	0.414
Δ eGFR (ml/min/1.73 m^2^)	− 1.16 [− 2.33, 0.02]	− 4.74 [− 7.83, − 1.6]	**0.035**

Analyses adjusted for age, BMI, sex, treatment group, number of comorbidities and the corresponding baseline laboratory parameter

**ɫ** p-PPI users vs. non-PPI users. Results in bold indicate statistically significant differences between p-PPI users and non-PPI users

*eGFR* estimated glomerular filtration rate, *non-PPI users* non-users of proton-pump inhibitors, *p-PPI users* persistent PPI users, *PTH* parathyroid hormone

#### Longitudinal Changes in Skeletal Characteristics as Assessed by HR-pQCT

**Fig. 2:**
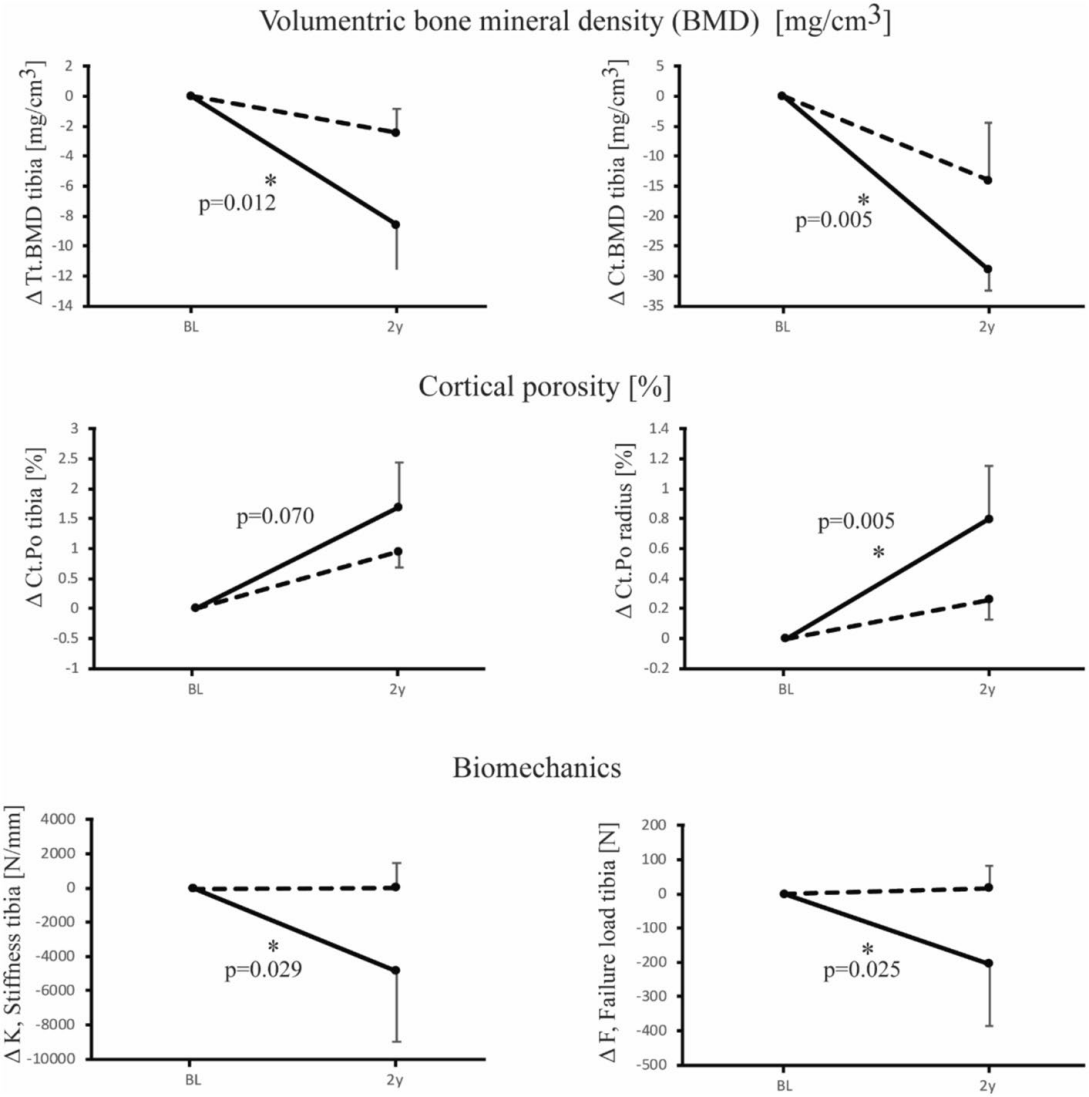
Plots of least square adjusted mean change (with 95% confidence interval) in distal tibial and distal radial HR-pQCT densitometric, microarchitectural and biomechanical parameters for p-PPI users (solid black line) and non-PPI users (dashed line). Depicted are results from Linear regression model 2. Asterisks indicate significant difference (p < 0.05) in absolute mean change between p-PPI users and non-PPI users. *Ct.BMD* volumetric BMD of the cortical compartment, *Ct.Po* intracortical porosity, *non-PPI users* non-users of proton pump inhibitors, *p-PPI users* persistent proton pump inhibitor users, *Tt.BMD* total volumetric bone mineral density (BMD) including trabecular and cortical bone

Unadjusted and adjusted absolute mean changes in volumetric bone mineral density, and bone microarchitectural and strength parameters over the 2-year follow-up period are tabulated for the distal tibia and radius in Table 4 and depicted in Fig. 2.

**Table 4:** Least square means of changes (Δ) in bone volumetric density, microarchitectural and strength parameters of the distal tibia and radius assessed via high-resolution peripheral quantitative computed tomography (HR-pQCT) over the 2-year study period in all study participants (*n* = 189), by proton-pump inhibitor (PPI) use

Distal tibia	Non-PPI users (*n* = 165)			P-PPI users (*n* = 24)			*p *values		
Bascic HR-pQCT measures	Model 0	Model 1	Model 2	Model 0	Model 1	Model 2	pǂ	p#	p°
Δ Tt.BMD [mg/cm^3^]	− 2.76 [− 4.5; − 1.1]	− 2.54 [− 4.2; − 0.9]	− 2.45 [− 4.1, − 0.9]	− 7.68 [− 12.4; − 3.0]	− 8.37 [− 12.9; 3.9]	− 8.58 [− 13.1, − 4.1]	*0.054*	**0.018**	**0.012**^**b**^
ΔTb.BMD [mg/cm^3^]	− 0.72 [− 1.6; 0.2]	− 0.61 [− 1.5; 0.3]	− 0.59 [− 1.5, 0.3]	− 1.30 [− 3.9; 1.3]	− 1.69 [− 4.2; 0.8]	− 1.68 [− 4.2, 0.8]	0.675	0.424	0.421
Δ Ct.BMD [mg/cm^3^]	− 14.56 [− 18.0; − 11.1]	− 14.20 [− 17.6; − 10.8]	− 14.07 [− 17.5, − 10.7]	− 26.93 [− 36.5; − 17.3]	− 28.4 [− 37.8; − 18.9]	− 28.96 [− 38.6, − 19.4]	**0.018**	**0.006**	**0.005**^**b**^
Δ Ct.Th [µm]	6.89 [− 4.6; 18.4]	7.30 [− 4.3; 18.9]	8.43 [− 3.0, 19.9]	− 13.94 [− 46.0; 18.1]	− 15.3 [− 47.8; 17.3]	− 19.49 [− 51.7, 12.7]	0.229	0.199	0.110
Δ Ct.Po [%]	0.96 [0.7; 1.2]	0.95 [0.7; 1.2]	0.95 [0. 7, 1.2]	1.64 [0.9; 2.4]	1.68 [0.9;2.4]	1.69 [0. 9, 2.4]	*0.090*	*0.071*	*0.070*
Δ Ct.PoDm [µm]	2.89 [0.4; 5.3]	2.80 [0.4; 5.3]	2.76 [0.31, 5.2]	2.38 [− 4.4; 9.2]	2.80 [− 4.1; 9.7]	3.09 [− 3.8, 9.9]	0.890	0.994	0.928
Δ Tb.N [mm^−1^]	0.03 [0.0; 0.1]	0.03 [0.0; 0.1]	0.03 [0.0, 0.1]	0.05 [− 0.0; 0.1]	0.05[− 0.0; 0.1]	0.06 [− 0.0, 0.1]	0.702	0.702	0.477
Δ Tb.Th [µm]	− 1.48 [− 2.5; − 0.4]	− 1.49 [− 2.5;− 0.4]	− 1.51 [− 2.5, − 0.5]	− 2.90 [− 5.8; 0.1]	− 2.86 [− 5.8; 0.0]	− 2.51 [− 5.3, 0.3]	0.362	0.384	0.508
Δ Tb.Sp [µm]	− 5.97 [− 12.8; 0.9]	− 6.29 [− 13.1; 0.6]	− 6.23 [− 12.8, 0.4]	− 15.16 [− 34.1; 3.8]	− 13.54 [− 32.7; 5.6]	− 16.5 [− 35.1, 2.0]	0.370	0.483	0.303

Biomechanics									
Δ Stiffness, K [N/mm]	− 40.94 [− 1495.3; 1413.4]	− 13.37 [− 1483.4; 1456.7]	20.69 [− 1445.9, 1487.3]	− 5136.92 [− 9154.6;− 1119.3]	− 5276.12 [− 9376.5; − 1175.8]	− 4848.08 [− 8957.8, − 738.4]	**0.020**	**0.019**	**0.029**
Δ E. Fail. Load, F [N]	13.31 [− 50.7; 77.3]	15.38 [− 49.2; 80.0]	16.72 [− 48.1, 81.6]	− 209.86 [− 386.7; − 33.0]	− 218.37 [− 398.6; − 38.2]	− 204.54 [− 386.3, − 22.8]	**0.020**	**0.017**	**0.025**^**b**^

Distal radius									
Bascic HR-pQCT measures	Model 0	Model 1	Model 2	Model 0	Model 1	Model 2	pǂ	p#	p°
Δ Tt.BMD [mg/cm^3^]	− 5.79 [− 7.89;− 3.7]	− 5.60 [− 7.6; − 3.6]	− 5.57 [− 7.5, − 3.6]	− 6.92 [− 12.5; − 1.3]	− 7.86 [− 13.2; − 2.5]	− 8.16 [− 13.5, − 2.8]	0.708	0.436	0.373
ΔTb.BMD [mg/cm^3^]	− 0.95 [− 1.9; − 0.0]	− 0.90 [− 1.8; 0.0]	− 0.91 [− 1.8, 0.0]	− 0.16 [− 2.8; 2.4]	− 0.37 [− 2.9; 2.1]	− 0.39 [− 2.9, 2.1]	0.559	0.695	0.698
Δ Ct.BMD [mg/cm^3^]	− 12.11 [− 15.7; − 8.5]	− 11.81 [− 15.2; − 8.4]	− 11.83 [− 15.3, − 8.4]	− 15.35 [− 24.9; − 5.8]	− 16.94 [− 26.2; − 7.7]	− 16.98 [− 26.4, − 7.6]	0.531	0.309	0.314
Δ Ct.Th [µm]	− 16.80 [− 25.7; − 7.9]	− 16.23 [− 25.0; − 7.5]	− 15.92 [− 24.7; − 7.2]	− 22.79 [− 46.6; 1.0]	− 26.25 [− 50.1; − 23.9]	− 28.22 [− 52.0, − 4.4]	0.642	0.439	0.342
Δ Ct.Po [%]	0.26 [0.1; 0.4]	0.26 [0.1; 0.4]	0.26 [0.1, 0.4]	0.79 [0.4; 1.1]	0.81 [0.5; 1.2]	0.80 [0.4,1.2]	**0.006**	**0.004**	**0.005**
Δ Ct.PoDm [µm]	0.97 [− 2.2; 4.2]	1.07 [− 2.2; 4.3]	0.97 [− 2.1, 4.1]	13.13 [4.5; 21.8]	12.56 [3.8, 21.3]	12.93 [4.56, 21.31]	**0.010**	**0.017**	**0.009**
Δ Tb.N [mm^−1^]	0.00 [− 0.0; 0.0]	0.00 [− 0.0;0.0]	− 0.00 [− 0.0, 0.0]	− 0.02 [− 0.1; 0.1]	− 0.02 [− 0.1; 0.1]	− 0.01 [− 0.1, 0.1]	0.636	0.713	0.857
Δ Tb.Th [µm]	− 0.53 [− 1.6; 0.6]	− 0.49 [− 1.6; 0.6]	0.44 [− 1.5, 0.6]	0.55 [− 2.4; 3.5]	0.35 [− 2.6, 3.3]	0.19 [− 2.6, 3.0]	0.496	0.602	0.681
Δ Tb.Sp [µm]	0.08 [− 8.4; 8.2]	0.08 [− 8.3; 8.5]	0.51 [− 7.6, 8.6]	10.20 [− 12.2; 32.6]	9.03 [− 13.7, 31.8]	6.10 [− 15.9, 28.1]	0.396	0.469	0.639

Biomechanics									
Δ Stiffness, K [N/mm]	− 1020.65 [− 1756.0; − 285.3]	− 962.67 [− 1660.6; − 264.8]	− 947.88 [− 1646.9, − 248.9]	− 360.59 [− 2333.8; 1612.6]	− 684.75 [− 2580.1; 1210.6]	− 665.44 [− 2563.3, 1232.4]	0.537	0.787	0.784
Δ E. Fail. Load, F [N]	− 41.51 [− 74.2; − 8.8]	− 38.79 [− 69.6; − 8.0]	− 38.18 [− 69.0, − 7.4]	− 4.54 [− 92.2; 83.2]	− 19.56 [− 103.1; 64.0]	− 18.21 [− 101.8, 65.4]	0.436	0.671	0.660

Data are presented as mean absolute changes **(**Δ) [95% CI]

Model 0: unadjusted analyses; Model 1: adjusted for age, BMI, and sex; Model 2: adjusted for age, BMI, sex, treatment group, number of comorbidities, and the corresponding BL bone parameter;

pǂ: comparing least square means derived from model 0 between p-PPI and non-PPI users. p#: comparing least square means derived from model 1 between p-PPI and non-PPI users

p°: comparing least square means derived from model 2 between p-PPI and non-PPI users. Results in bold indicate statistically significant differences between p-PPI and non-PPI users

^b^*p* < 0.05 after adjustment for age, BMI, sex, treatment group, number of comorbidities, and the corresponding BL bone parameter and after correcting for multiple comparisons using the hierarchical false discovery rate (FDR) method with an FDR set to 0.05

*BL* baseline, *BMI* body mass index, *BMD* volumetric bone mineral density, *Ct* cortical, *Ct.Po* intracortical porosity, *Ct.PoDm* mean cortical pore diameter, *Ct.Th* cortical thickness, *E. Fail. Load* estimated failure load, *p-PPI users* persistent PPI users, *Tb* the trabecular, *Tb.N* trabecular number, *Tb.Sp* trabecular separation, *Tb.Th* trabecular thickness, *Tt* total

During the 2-year follow-up, p-PPI users lost about 3.5 times more total volumetric bone mineral density (ΔTt.BMD p-PPI group: − 8.58 mg/cm^3^ [− 13.1, − 4.1; 95% CI] vs. non-PPI group: − 2.45 mg/cm^3^ [− 4.1, − 0.9; 95% CI], *p* = 0.012), and about twice as much cortical volumetric BMD (ΔCt.BMD − 28.96 mg/cm^3^ [− 38.6, − 19.4; 95% CI] vs. − 14.07 mg/cm^3^ [− 17.5, − 10.7; 95% CI], *p* = 0.0047) relative to non-PPI users at the distal tibia, while the trabecular volumetric BMD remained unchanged in both groups (Table 4, model 2). With respect to biomechanical indices, p-PPI users demonstrated a significantly larger reduction in bone strength indices relative to non-PPI users over the 2-year follow-up: this included a significant decline in bone stiffness K (Table 4, model 2: ΔK − 4848.1 N/mm [95% CI: − 8957.8, − 738.4;], vs. 20.7 N/mm [− 1445.9, 1487.3; 95% CI], *p* = 0.029), as well as a significant decrease in estimated failure load F (ΔF − 204.5 N [− 386.3, − 22.8, 95% CI] vs. 16.7 N [− 48.1, 81.6; 95% CI], *p* = 0.025). These significant reductions in mean cortical BMD and in biomechanical indices in the p-PPI relative to the non-PPI group persisted in the unadjusted linear regression models (model 0) or models adjusted for age, sex, and BMI (model 1).

At the distal radius, significant changes in cortical bone microarchitecture were observed in the p-PPI group relative to non-PPI users and changes remained significant and of similar magnitude throughout all three regression models: p-PPI users exhibited a significant, almost three times larger increase in cortical porosity and an about 13 times larger significant increase in average cortical pore diameter compared to the non-PPI group over time (Table 4, model 2: Δ Ct.Po at the distal radius: 0.80 [0.4, 1.2; 95% CI] vs. 0.26 [0.1, 0.4; 95% CI], *p* = 0.005) and cortical pore diameter (Δ Ct.PoDm 12.9 μm [4.6, 21.3; 95% CI] vs. 0.97 μm [− 2.1, 4.1; 95% CI], *p* = 0.009). At the distal tibia, a statistical trend toward a higher (intra)cortical porosity increase was also present in the p-PPI group (*p* = 0.070), which became significant in a sensitivity analysis among participants with and without PPI use who were free of knee pain at the non-operated, HR-pQCT-measured leg at the 2-year visit (Δ Ct. Po distal tibia: 1.79 [1.1; 2.5, 95% CI] vs. 0.67 [0.4;0.9, 95% CI]; *p* = 0.003, Supplemental Table B). Similar to the tibial site, the decrease in total and cortical volumetric BMD was numerically higher in the p-PPI group relative to non-PPI users at the distal radius; however, none of the changes translated into statistical significance.

After correcting for multiple comparisons using the hierarchical FDR method [35], the decrease in total vBMD at the distal tibia and the decrease in cortical vBMD at the distal tibia remained statistically significantly larger in the p-PPI vs. non-PPI users (p < 0.05). In addition, the decrease in estimated failure load F at the tibial site also remained significantly larger in the p-PPI vs. non-PPI group (*p* < 0.05), while the significances in Δ stiffness K at the distal tibia and in Δ cortical porosity and Δ porosity diameter at the radius were lost.

At both distal scan sites, no changes in trabecular microarchitectural parameters, such as trabecular number, thickness, and spacing, were noted for either of the groups during follow-up.

#### Sensitivity Analysis of Longitudinal Skeletal Changes

In a sensitivity analysis, we examined the robustness of longitudinal HR-pQCT-derived skeletal findings (which had been acquired at the non-operated leg) when including only those study participants with and without PPI use who self-reported no pain at the non-operated knee at the 2-year visit (WOMAC pain score of 0, Supplemental Tables B and C). Demographically, this subset of participants exhibited similar age and BMI means as participants in the main analyses (*p* > 0.05), and displayed normal to minimally reduced performance and knee function as indicated by their average WOMAC functional scores ranging from 0.77 ± 1.5 to 1.28 ± 2.3 in the p-PPI and non-PPI groups, respectively. With respect to skeletal characteristics, we noted that these sensitivity analyses had very little impact on the overall reported significances of the distal tibial and distal radial bone parameters. Specifically, mean absolute changes in cortical BMD and in biomechanical indices at the distal tibia and in cortical porosity and cortical pore diameter at the distal radius remained significant, supporting our findings in the main analyses.

## Discussion

To the best of our knowledge, this is the first study that used HR-pQCT to longitudinally explore the association of PPI use and bone health at the bone microarchitectural level.

Our findings suggest that adults aged 60 years and older with a regular PPI intake on more than 50% of days over a 2-year duration exhibited a significant decline in total and cortical volumetric bone mineral density at the distal tibia compared to non-PPI users. Regarding the clinical relevance of these findings, the distal tibial bone mineral density changes observed for PPI users in our study, unselected for osteoporosis, appeared to be of a similar magnitude as documented in a randomized double-blind trial using the same HR-pQCT method for postmenopausal women taking 70 mg alendronate orally for 1 year [38]. Also, when looking at the annual cortical BMD loss detected in the PPI user group, this annual loss was approximately two times greater than the annual cortical BMD change reported in a RCT for postmenopausal women receiving 60 mg denosumab subcutaneously every 6 months for 1 year [38]. These clinical comparisons suggest that PPI users in our study experienced annual total and cortical BMD losses large enough to be of clinical relevance. Additionally, our findings build on and extend on two previous DXA-based central skeletal studies which reported a significant reduction in DXA-based hip T-scores after 1-year of PPI usage [9] and found a significantly lower hip and spine DXA-based bone mineral content in patients who had taken PPIs for about 6.7 years [39] prior to the study visit. At first glance contradictory to our results, Targownik et al. [40] did not find any association between PPI use and changes in DXA-based areal BMD over 5–10 years. However, areal BMD was measured in Targownik’s study at central skeletal sites such as at the femoral neck, total hip, and lumbar spine, while our study examined PPI-associated bone health on the peripheral skeleton. In addition, contrary to our study, in which PPI medication usage was tracked continuously over the full 24 month study period, PPI use was only queried in Targownik’s study snapshot-like at 3 distinct time points (baseline, 5-year and 10-year follow-up) and it was not known if participants had been taking any PPIs during the 5-year time intervals between visits. Therefore, participants might have been classified as PPI users, who might have been using PPIs only for a very short time or discontinuously. Due to these reasons, the measured areal BMD change in Targonik’s study may not accurately reflect the association between PPI and BMD.

Another important finding of our study was that regular PPI users exhibited a significant greater decline in distal tibia calculated bone strength during the 2-year follow-up period than non-PPI users. As bone strength is regarded as an important predictor of bone fracture, our findings of declining bone strength in PPI users substantiate previous reports linking PPI usage to a higher risk of fragility fracture [41,42]. Apart from our longitudinal study, there has been only one other study by Targownik et al. examining the relationship between bone strength and PPI usage to date. That cross-sectional study utilized 3D-QCT of the femur to derive buckling ratio and section modulus as geometry-based estimates of bone strength [22] and failed to find an association between bone strength estimates and PPI intake. The lack of differences in bone strength may either be attributed to the cross-sectional study design or to the geometry-based parameters such as buckling ratio and section modulus which are not comparable to the in our study utilized HR-pQCT-derived bone strength analysis via micro-finite-element modeling (FEA).

At the distal radius, we detected a significantly larger increase in the amount and diameter of cortical pores in the p-PPI-group relative to non-PPI users. Previous biomechanic and cadaveric studies have identified and substantiated cortical porosity as a major determinant of bone strength, stiffness, and fracture toughness [43–46]. More importantly, the cortical porosity of the inner transitional zone—also measured via a first-generation HR-pQCT scanner—was found to predict fragility fractures in postmenopausal women [47]—underscoring the relevance of cortical porosity as an important cortical microarchitectural HR-pQCT-derived parameter. Due to Limitations in image resolution inherent to HR-pQCT scanners of the first generation, cortical porosity in our study could only be spatially resolved to pores larger than 100 μm in diameter. Therefore, only changes in cortical “macro-porosity” were detectable, while changes in micropores that might represent osteocyte lacunae or canaliculi or smaller Haversian canals could not be resolved unless they had grown to a resolvable size (> 100 μm). In light of the limitations in spatial image resolution of our first-generation HR-pQCT scanner, it remains unclear, how and if PPI use is associated with changes in cortical porosity at the subresolution microporosity level. However, given that bone´s mechanical competence degrades particularly with increasing cortical pore size [48], most likely explained by reducing the amount of available bone matrix area for microcrack propagation [49] and by facilitating microcrack initiation through larger stress concentrations [50], our (macro)cortical porosity measurement Likely captures the most relevant increases in cortical porosity from a biomechanical point of view. Further high-resolution in vivo or ex vivo studies are needed to assess PPI-induced cortical porosity changes below the current resolution of 100 μm.

The fact that we did not observe a uniform pattern of bone changes at both skeletal sites could indicate that PPI-related bone loss may take place in a site-specific manner, and may be occurring predominantly at weight bearing bones such as the tibia—an effect that has been observed in other osteoporotic [51,52] and lactation studies [53]. More research is needed to further understand the regulation of location-specific patterns of bone loss.

With respect to laboratory measures, we detected a small, yet significant decline in estimated glomerular filtration rate in the p-PPI group over the 2 year study period along with a very small significant increase in serum calcium levels. While we consider the change in calcium level as too small and too spurious to allow for further interpretation, an increasing body of literature is linking PPI use with adverse kidney outcomes [54], such as, e.g., acute kidney injury [55] or progression of CKD [56]. Interestingly, risk of PPI-induced kidney damage was found to gradually increase with higher dosages and prolonged intake of PPIs [57], particularly in individuals ≥ 70 years old [58]. As intake of non-steroidal anti-inflammatory drugs (NSAIDs), which are considered nephrotoxic drugs [59], was also slightly higher in the p-PPI group, the observed significant decline in eGFR in the p-PPI group may likely be multifactorial resulting from a combination of an age-related physiological decline in eGFR [60], potentially coupled with PPI- and/or NSAID-mediated renal nephrotoxic effects.

The potential biological mechanisms through which PPIs may drive bone loss and impact bone strength remain unclear. Possible theories involve PPI-induced changes in the gastrointestinal-bone axis particularly in the gastrointestinal microbiome and an impaired calcium absorption. Recent findings suggests that oral PPI intake results in significant alterations of the gut microbiota composition with significant decreases in gut bacterial alpha diversity and changes of 20% of bacterial gut taxa [61]. In addition, emerging evidence from rodent studies provides first hints that disruption of the gut microbiome reduces cortical BMD and impairs bone strength [62], while administration of probiotics improves bone mineral density and bone strength in ovariectomized rats [63]. In line with those findings, latest clinical studies suggest modest associations between altered gut microbiota and BMD [64,65] in osteoporotic individuals. Apart from the changes in the microbiome-bone axis, the oral intake of PPIs may also affect gastric acidification and via an increase in gastric pH reduce intestinal calcium absorption [66], resulting in blood hypocalcemia. This in turn may trigger (by means of secondary hyperparathyroidism) a compensatory release of calcium from the bones eventually decreasing bone mineral density and strength. Supporting evidence for this theory has been published by O´Connell et al. who demonstrated in a randomized, double-blind, cross-over clinical trial that oral intake of omeprazole significantly reduced gastric calcium carbonate absorption compared to placebo pill intake [67] and is also in line with the rodent work reported by Schinke et al. [68]. Of note, in our study, calcium serum levels in the p-PPI group did not decrease over the 2-year study period and were accompanied by a small increase in PTH serum levels (within the normal limits of PTH reference ranges), and thus not suggestive of secondary hyperparathyroidism. Therefore, it seems less likely that a PPI-induced impairment of gastric acidification may be the main pathomechanism of the observed bone deterioration. Other mechanisms such as a PPI-mediated increase in osteoblastic/osteoclastic apoptotic rate or, e.g., a decreased collagen type 1 expression might be also at play [8]. However, additional studies are needed to validate those findings on a tissue level.

Whether the decline in volumetric BMD and bone strength at the tibia may be influenced by a greater proportion of participants with concomitant NSAIDs usage in the PPI user group is unclear as there are currently no longitudinal HR-pQCT or pQCT studies available examining the influence of NSAIDs on bone microarchitecture. However, previous cross-sectional studies have reported higher areal BMDs by DXA in NSAID users [69] than in non-NSAID users even after correcting for osteoarthritis and even at skeletal sites not affected by the local changes related to osteoarthritis [70]. In addition, significantly higher volumetric BMD of the trabecular and cortical compartment of the L3 vertebra as measured via quantitative computed tomography of the spine were found in NSAID users compared to non-users [71]. A large prospective study found a small increase in spine and whole body aBMD by DXA over a 10-year follow-up in NSAID versus non-NSAID users but observed an increased fracture risk in NSAID users which may be a result from central nervous effects of NSAIDs on postural balance [72]. Given that current clinical evidence reports either a moderate beneficial effect of NSAIDs on BMD or no effect of NSAIDs on BMD, we feel confident that our current significant findings of declining BMD and bone strength in PPI users represent true findings and are not relevantly influenced by concomitant NSAID usage. Based on the current literature, it is possible that the range of decline in BMD and bone strength measured in our PPI users may actually be larger, due to the potential counteracting beneficial effect of concomitant NSAID usage on BMD. Large clinical trials on the effect of NSAID usage on bone microarchitecture are needed to explore this in more detail.

Our study has several strengths. First, this study was prospective by design and had a relatively long follow-up period of 2 years. In addition, it employed HR-pQCT for the first time in the context of PPI and bone health and, thus, a high-resolution imaging modality that allows not only for cross-sectional but also longitudinal detailed quantification of human bone microarchitecture and strength at a resolution of 82 µm isotropic voxel size. Another strength of our study is that bone strength indices, such as stiffness and failure load, were modeled using micro-finite-element analysis (µFEA), a method which has been proven to better predict bone strength than any DXA-based parameter [73].

Our study has several limitations. First, although we used data from a randomized-controlled vitamin D trial, this study was solely designed as a prospective observational study, and not as a RCT with PPI intake as intervention. Thus, our results are purely associative in nature and do not allow to draw any causal inferences between PPI usage and bone health. Second, the sample size in the p-PPI user group was relatively small which made further subgroup analyses impossible. Moreover, the proportion of individuals in the DXA range of osteoporosis included in our study was rather low, and therefore, our results may not be generalizable to patient cohorts with high rates of osteoporosis. Moreover, we did not have DXA-based areal BMD changes available. As another limitation, we would like to acknowledge that multiple HR-pQCT parameters were tested in this study, increasing the potential for false positives (type 1 error). If the most conservative correction method for multiple testing—which is the Bonferroni method—is applied, none of the changes in HR-pQCT parameters would be significant due to the large number of parameters being tested. However, the Bonferroni method runs the risk of overcorrecting data and does not account for a hierarchical data structure as present with HR-pQCT data. We therefore applied in accordance with the HR-pQCT guidelines [36] and alternate multiple correction method, namely the hierarchical False Discovery Rate controlling method by Yekutieli [35], which is based on the Benjamini and Hochberg false discovery rate controlling procedure [74] but accounts additionally for the hierarchical structure of data. Using this multiple comparison correction method, the main significances of change in total vBMD, and change in cortical vBMD and in failure load at the distal tibia persisted even after correcting for multiple comparisons. Another Limitation of our study pertains to the fact that documentation of type and timeline of PPI usage was based exclusively on patient self-report which may have introduced some bias. In addition, we did not have any information on exact PPI doses. HR-pQCT image registration was performed in our study using a 2D image registration technique and not via 3D rigid registration, which could account for potential angular image differences [36]. Nevertheless, the 2D image registration method is considered as the standard registration technique and has been validated in longitudinal HR-pQCT studies [33,75] and is therefore an integral part of the current manufacturer’s standard image postprocessing software.

In summary, our results suggest that regular PPI usage of more than 50% of time over a 2-year period is associated with a significant deterioration of volumetric bone mineral density and bone strength at the distal tibia and with a significant decline in cortical microarchitecture at the distal radius in older patients. Given the widespread use of PPIs worldwide, particularly in older adults where PPIs are often used chronically without clear therapeutic intent, our current findings highlight the need for clinicians to carefully scrutinize indications for PPI prescriptions in older patients, minimize exposure time to PPIs, and to de-prescribe PPIs when there is no reason to continue treatment.

## Supplementary Information

Below is the link to the electronic supplementary material.

Supplementary file1 (DOCX 50 KB)
