# Supplementary material for: Regular Proton-Pump Inhibitor Intake is Associated with Deterioration of Peripheral Bone Mineral Density, Microarchitecture, and Strength in Older Patients as Assessed by High-Resolution Peripheral Quantitative Computed Tomography (HR-pQCT)
Source: Calcif Tissue Int. 2025 Oct 22;116(1):131. doi: 10.1007/s00223-025-01420-7 (PMC12546302; doi:10.1007/s00223-025-01420-7)
Supplement: Supplementary file 1 — Supplementary file1 (DOCX 50 KB) [file 223_2025_1420_MOESM1_ESM.docx]

Supplemental Material

*High-resolution peripheral quantitative computed tomography (HR-pQCT) imaging*

At baseline and at 2-year follow-up, patients underwent high-resolution peripheral quantitative computed tomography (HR-pQCT) scanning at the standard distal tibial and radial scan regions. All scans were performed using the same clinical HR-pQCT system manufactured by Scanco Medical AG (XtremeCT I, Scanco Medical AG, Brüttisellen, Switzerland). A well-established in-vivo standard imaging protocol with 60 kVP, 900 μA, 100 ms integration time, 126-mm field of view as imaging parameters was used ^(1)^. In general, the tibia of the non-operated leg and radius of the non-dominant hand was scanned. In case the patient reported a history of fracture at the non-dominant tibia or radius, the contralateral side was scanned. For the scans, the patient´s lower extremity was immobilized in a carbon fiber cast and anchored in the scanner to minimize motion artifacts. A single anteroposterior scout radiograph was obtained to ensure correct positioning of the tomographic scan volume. A reference line was first placed at the apex of the distal articular plateau of the tibia according to the guidelines of the manufacturer and the scan region was set based on the scout scan using a fixed offset of 22.5 mm proximal to this reference line. A total distal tibial scan volume of 9.02 mm in length (110 slices) proximal of the offset was then acquired. For each scan, 750 projections were obtained, and the effective patient dose totaled approximately 3 μSv. The average time per scan was 2.8 minutes. Images were reconstructed to a 1536 x 1536 matrix, allowing for a final nominal resolution of 82 μm isotropic voxels. To calculate densitometric bone parameters, image attenuation values were calibrated against the attenuation values derived from a standardized hydroxyapatite (HA) phantom that was scanned daily for quality assurance^(2)^.

*HR-pQCT image segmentation and registration*

All images were contoured semi-automatically by four trained professionals (AS, MB, UH, UM), according to a standard protocol provided by Scanco and contoured images were analyzed as described previously ^(3)^. Prior to contouring and to account for motion artifacts, all scans were visually scored for presence and severity of motion artifacts using the grading scheme developed by Pialat et al.^(4)^.To ensure spatial correspondence between baseline and follow-up images the manufacturer’s standard longitudinal registration algorithm was employed to all baseline and follow-up images, a method which has already been successfully used and validated in many longitudinal clinical trials ^(5,6)^. In this 2D registration technique, scans are matched based on total cross-sectional area and the cortical region is defined independently in the baseline and the follow-up scans.

*Standard image analysis*

All HR-pQCT images were then further analyzed using the same, well-established standard image evaluation protocol ^(5,7)^. This protocol retrieves first the periosteal perimeter through a semi-automated edge-finding algorithm which generates a closed contour around the periosteal surface. It then extracts the cortical compartment volume of Interest (VOI) by using a smoothening Gaussian operator followed by a fixed threshold and calculates cortical bone parameters such as the total volumetric bone mineral density (vBMD), cortical vBMD (Ct.vBMD), and cortical area (Ct.Ar). Additionally, trabecular parameters (trabecular number [Tb.N], trabecular thickness [Tb.Th], trabecular separation [Tb.Sp]) are computed using the methods described by Burghardt and colleagues ^(6)^.

*Micro-finite-element (µFE) analysis*

In order to compute the apparent biomechanical properties, standard linear micro-finite-element analysis (µFEA) modeling using high friction axial (superior-inferior) compression, was performed for each ultra-distal scan site under 1% of strain as described previously ^(6)^. For all simulations and all bone elements, homogeneous mechanical properties were assumed, and a mesh of isotropic brick elements was generated from the binary image. Each element was assigned an elastic modulus of 10 GPa and a Poisson's ratio of 0.3 and reaction forces were calculated at the proximal and distal ends of the scan region for the prescribed displacements via an iterative solver (Scanco FE Software, Version 1.12, Scanco Medical). All microfinite element computations were carried out at the Center of Aging and Mobility. No models had to be excluded due to convergence limitations. For each site, the biomechanical indices stiffness *K* and the failure load *F* were calculated. The failure load was calculated as the force for which more than 2% of the tissue would experience a strain beyond 0.7%^(8)^.

**Supplemental Results**

**Supplemental Table A**

| **Supplemental Table A** | **Overall** | **Non-PPI users** | **p-PPI users** | **p-values ɫ** |
| --- | --- | --- | --- | --- |
| **WOMAC function score (score 0-100)** | **(n=189)** | **(n=165)** | **(n=24)** |  |
| Operated knee: |  |  |  |  |
| Functional difficulties at baseline visit | 25.3 ± 13.7 | 25.2 ± 13.6 | 26.2 ± 14.2 | 0.736 |
| Functional difficulties at 24 months visit | 6.8 ± 9.6 | 6.4 ± 9.4 | 9.4 ± 11.0 | 0.159 |
| P values across time |  | **<0.001** | **<0.001** |  |
| Non-operated knee: |  |  |  |  |
| Functional difficulties at baseline visit | 4.0 ± 8.2 | 4.0 ± 8.2 | 3.6 ± 8.1 | 0.830 |
| Functional difficulties at 24 months visit | 4.4 ± 7.7 | 4.1 ± 7.1 | 5.8 ± 10.9 | 0.330 |
| P values across time (paired t test) |  | 0.794 | 0.153 |  |
|  |  |  |  |  |
| Data are expressed as mean ± SD unless otherwise stated **ɫ** p-PPI users vs. non-PPI users.  Abbreviations: non-PPI users, non-users of proton pump inhibitors; p-PPI users, persistent PPI users; WOMAC, Western Ontario and McMaster Universities Arthritis Index | | | | |

*Supplemental Table A legend: Baseline and 24 months Western Ontario and McMaster Universities Arthritis Index (WOMAC) function scores ranging from 0 to 100 shown for all study participants and for non-PPI and p-PPI users separately. A WOMAC function score of 0 indicates no impairment in physical knee function during daily activities. To compare means across time points, paired- t-tests were performed.*

| **Supplemental Table B**. Unadjusted mean absolute changes **(Δ)** in distal tibial volumetric bone mineral density, microarchitectural and strength parameters as assessed at the non-operated leg via HR-pQCT over the 2-year study period in all study participants (n=189), by PPI use, and for all those study participants who were free of any knee pain (WOMAC pain score of 0) at the non-operated leg at the 2-year study visit (n=119), by PPI use (sensitivity analysis, right half of the table). Significant p-values are printed in bold print. | | | | | | |
| --- | --- | --- | --- | --- | --- | --- |
|  | **All study participants** | | | **Study participants without knee pain at the non-operated leg**  **at the 2-year visit** | | |
|  | **Non-PPI users**  **(N=165)** | **p-PPI users**  **(n=24)** | **p-values**  **ɫ** | **Non-PPI users**  **(n=104)** | **p-PPI users**  **(n=15)** | **p-values ɫ** |
| ***Patient characteristics*** | mean ± SD | mean ± SD |  | mean ± SD | mean ± SD |  |
| Age [years] | 70.7 ± 6.7 | 71.4 ± 6.1 | 0.644 | 70.3 ± 6.8 | 70.2 ± 6.5 | 0.951 |
| BMI [kg/m^2^] | 27.0 ± 3.9 | 29.3 ± 4.0 | **0.008** | 27.0 ± 3.7 | 28.8 ± 4.3 | *0.093* |
| **Δ** BMI over 24 months [kg/m^2^] | 0.6 ± 1.3 | 0.6 ± 1.6 | 0.891 | 0.5 ± 1.4 | 0.1 ± 1.6 | 0.333 |
| Treatment arm  Vit D 800 IU/daily [%] | 52.1 | 50 | 0.846 | 56.7 | 40 | 0.224 |
| WOMAC function score at non-operated knee (0-100) at 24 months | 4.1 ± 7.1 | 5.8 ± 10.9 | 0.330 | 1.28 ± 2.3 | 0.77 ± 1.5 | 0.267 |
| ***Distal Tibia***  ***Basic HR-pQCT measures*** | **Mean absolute**  **change (Δ) [95% CI]** | **Mean absolute**  **change (Δ) [95% CI]** |  | **Mean absolute**  **change (Δ) [95% CI]** | **Mean absolute**  **change (Δ) [95% CI]** |  |
| Δ Tt.BMD [mg/cm^3^] | -2.76 [-4.5; -1.1] | -7.68 [-12.4; -3.0] | *0.054* | -2.78 [-5.0; -0.59] | -8.34 [-14.0; -2.7] | *0.071* |
| ΔTb.BMD [mg/cm^3^] | -0.72 [-1.6; 0.2] | -1.30 [-3.9; 1.3] | 0.675 | -1.28 [-2.6; 0.0] | -0.71 [-4.0; 2.6] | 0.748 |
| Δ Ct.BMD [mg/cm^3^] | -14.56 [-18.0; -11.1] | -26.93 [-36.5; -17.3] | **0.018** | -13.50 [-17.6; -9.4] | -28.92 [-39.4; -18.4] | **0.008** |
| Δ Ct.Th [µm] | 6.89 [-4.6; 18.4] | -13.94 [-46.0; 18.1] | 0.229 | 9.13 [- 6.2; 24.5] | -21.88 [ -61.6; 17.8] | 0.152 |
| Δ Ct.Po [%] | 0.96 [0.7; 1.2] | 1.64 [0.9; 2.4] | *0.090* | 0.67 [0.4; 0.9] | 1.79 [1.1; 2.5] | **0.003** |
| Δ Ct.PoDm [µm] | 2.89 [0.4; 5.3] | 2.38 [-4.4; 9.2] | 0.890 | 1.54 [-1.4; 4.5] | 2.74 [-4.9; 10.4 ] | 0.774 |
| Δ Tb.N [mm^-1^] | 0.03 [0.0; 0.1] | 0.05 [-0.0; 0.1] | 0.702 | 0.02 [-0.0; 0.6] | 0.07 [-0.0; 0.2] | 0.343 |
| Δ Tb.Th [µm] | -1.48 [-2.5; -0.4] | -2.90 [-5.8; 0.1] | 0.362 | -1.33 [-2.8; 0.1] | -3.50 [-7.2; 0.2] | 0.282 |
| Δ Tb.Sp [µm] | -5.97 [-12.8; 0.9] | -15.16 [-34.1; 3.8] | 0.370 | - 3.12 [- 12.6; 6.4] | - 20.43 [-44.9; 4.0] | 0.193 |
| ***Biomechanics*** |  |  |  |  |  |  |
| Δ Stiffness, K [N/mm] | -40.94 [-1495.3; 1413.4] | -5136.92 [-9154.6;-1119.3] | **0.020** | 1.39 [-1906.1; 1908.9] | -5929.52 [-10846.0; -1013.1] | **0.028** |
| Δ Estimated Failure Load, F [N] | 13.31 [-50.7; 77.3] | -209.86 [-386.7; -33.0] | **0.020** | 10.71 [-71.6; 93.0] | -249.56 [-461.7; -37.4] | **0.025** |
| **ɫ** persistent PPI users vs. non-PPI users  Abbreviations: Ct.BMD, volumetric BMD of the cortical compartment; Ct.Po, intracortical porosity; Ct.PoDm, mean cortical pore diameter; Ct.Th, cortical thickness; HR-pQCT, high resolution peripheral quantitative computed tomography; non-PPI users, non-users of proton pump inhibitors; p-PPI users, persistent PPI users; Tb.BMD, volumetric BMD of the trabecular compartment; Tb.N, trabecular number; Tb.Sp, trabecular separation; Tb.Th, trabecular thickness; Tt.BMD, total volumetric bone mineral density including trabecular and cortical bone. | | | | | | |

| **Supplemental Table C**. Unadjusted mean absolute changes **(Δ)** in distal radial volumetric bone mineral density, microarchitectural and strength parameters as assessed at the non-operated leg via HR-pQCT over the 2-year study period in all study participants (n=189), by PPI use, and for all those study participants who were free of any knee pain (WOMAC pain score of 0) at the non-operated leg at the 2-year study visit (n=119), by PPI use (sensitivity analysis, right half of the table). Significant p-values are printed in bold print. | | | | | | |
| --- | --- | --- | --- | --- | --- | --- |
|  | **All study participants** | | | **Study participants without knee pain at the non-operated leg**  **at the 2-year visit** | | |
|  | **Non-PPI users**  **(N=165)** | **p-PPI users**  **(n=24)** | **p-values**  **ɫ** | **Non-PPI users**  **(n=104)** | **p-PPI users**  **(n=15)** | **p-values ɫ** |
| ***Patient characteristics*** | mean ± SD | mean ± SD |  | mean ± SD | mean ± SD |  |
| Age [years] | 70.7 ± 6.7 | 71.4 ± 6.1 | 0.644 | 70.3 ± 6.8 | 70.2 ± 6.5 | 0.951 |
| BMI [kg/m^2^] | 27.0 ± 3.9 | 29.3 ± 4.0 | **0.008** | 27.0 ± 3.7 | 28.8 ± 4.3 | *0.093* |
| **Δ** BMI over 24 months [kg/m^2^] | 0.6 ± 1.3 | 0.6 ± 1.6 | 0.891 | 0.5 ± 1.4 | 0.1 ± 1.6 | 0.333 |
| Treatment arm  Vit D 800 IU/daily [%] | 52.1 | 50 | 0.846 | 56.7 | 40 | 0.224 |
| WOMAC function score at non-operated knee (0-100) at 24 months | 4.1 ± 7.1 | 5.8 ± 10.9 | 0.330 | 1.28 ± 2.3 | 0.77 ± 1.5 | 0.267 |
| ***Distal Radius***  ***Basic HR-pQCT measures*** | **Mean absolute**  **change (Δ) [95% CI]** | **Mean absolute**  **change (Δ) [95% CI]** |  | **Mean absolute**  **change (Δ) [95% CI]** | **Mean absolute**  **change (Δ) [95% CI]** |  |
| Δ Tt.BMD [mg/cm^3^] | -5.79 [-7.89;-3.7] | -6.92 [-12.5; -1.3] | 0.708 | - 4.67 [-7.1; -2.2] | - 7.62 [-13.9; -1.3] | 0.387 |
| ΔTb.BMD [mg/cm^3^] | -0.95 [-1.9; -0.0] | -0.16 [-2.8; 2.4] | 0.559 | -0.94 [-2.2; 0.3] | - 0.51 [- 3.7; 2.7] | 0.808 |
| Δ Ct.BMD [mg/cm^3^] | -12.11 [-15.7; -8.5] | -15.35 [-24.9; -5.8] | 0.531 | -9.65 [-13.9; -5.4] | - 18.40 [-29.5; -7.3] | 0.148 |
| Δ Ct.Th [µm] | -16.80 [-25.7; -7.9] | -22.79 [-46.6; 1.0] | 0.642 | - 7.47 [- 16.9; 2.0] | -15.75 [- 40.1; 8.6] | 0.531 |
| Δ Ct.Po [%] | 0.26 [0.1; 0.4] | 0.79 [0.4; 1.1] | **0.006** | 0.28 [0.1; 0.4] | 0.93 [0.5; 1.4] | **0.005** |
| Δ Ct.PoDm [µm] | 0.97 [-2.2; 4.2] | 13.13 [4.5; 21.8] | **0.010** | 0.40 [- 3.8; 4.6] | 15.91 [5.1; 26.7] | **0.009** |
| Δ Tb.N [mm^-1^] | 0.00 [-0.0; 0.0] | -0.02 [-0.1; 0.1] | 0.636 | 0.00 [-0.0; 0.0] | - 0.01 [-0.1; 0.1] | 0.802 |
| Δ Tb.Th [µm] | - 0.53 [-1.6; 0.6] | 0.55 [-2.4; 3.5] | 0.496 | -0.56 [- 1.9; 0.8] | 0.1 [-3.4; 3.5] | 0.738 |
| Δ Tb.Sp [µm] | 0.08 [-8.4; 8.2] | 10.20 [-12.2; 32.6] | 0.396 | -1.79 [-12.5; 8.9] | 9.93 [- 17.6; 37.5] | 0.434 |
| ***Biomechanics*** |  |  |  |  |  |  |
| Δ Stiffness, K [N/mm] | -1020.65 [-1756.0; -285.3] | -360.59 [-2333.8; 1612.6] | 0.537 | -715.52 [-1612.1; 181.06] | -362.78 [-2673,6; 1948.0] | 0.778 |
| Δ Estimated Failure Load, F [N] | -41.51 [-74.2; -8.8] | -4.54 [-92.2; 83.2] | 0.436 | -28.35 [-68.5, 11.7] | -5.78 [-109.1; 97.6] | 0.687 |
| **ɫ** persistent PPI users vs. non-PPI users  Abbreviations: Ct.BMD, volumetric BMD of the cortical compartment; Ct.Po, intracortical porosity; Ct.PoDm, mean cortical pore diameter; Ct.Th, cortical thickness; HR-pQCT, high resolution peripheral quantitative computed tomography; non-PPI users, non-users of proton pump inhibitors; p-PPI users, persistent PPI users; Tb.BMD, volumetric BMD of the trabecular compartment; Tb.N, trabecular number; Tb.Sp, trabecular separation; Tb.Th, trabecular thickness; Tt.BMD, total volumetric bone mineral density including trabecular and cortical bone. | | | | | | |

**References:**

1. Boutroy S, Bouxsein ML, Munoz F, Delmas PD. In vivo assessment of trabecular bone microarchitecture by high-resolution peripheral quantitative computed tomography. J. Clin. Endocrinol. Metab. 2005 Dec;90(12):6508–15.

2. Cheung AM, Adachi JD, Hanley DA, Kendler DL, Davison KS, Josse R, Brown JP, Ste-Marie L-G, Kremer R, Erlandson MC, Dian L, Burghardt AJ, Boyd SK. High-Resolution Peripheral Quantitative Computed Tomography for the Assessment of Bone Strength and Structure: A Review by the Canadian Bone Strength Working Group. Curr Osteoporos Rep. 2013 Jun 1;11(2):136–46.

3. Burghardt AJ, Buie HR, Laib A, Majumdar S, Boyd SK. Reproducibility of direct quantitative measures of cortical bone microarchitecture of the distal radius and tibia by HR-pQCT. Bone. 2010 Sep 1;47(3):519–28.

4. Pialat JB, Burghardt AJ, Sode M, Link TM, Majumdar S. Visual grading of motion induced image degradation in high resolution peripheral computed tomography: Impact of image quality on measures of bone density and micro-architecture. Bone. 2012 Jan 1;50(1):111–8.

5. Laib A, Häuselmann HJ, Rüegsegger P. In vivo high resolution 3D-QCT of the human forearm. Technology and Health Care. IOS Press; 1998 Jan 1;6(5–6):329–37.

6. Burghardt AJ, Kazakia GJ, Sode M, de Papp AE, Link TM, Majumdar S. A longitudinal HR-pQCT study of alendronate treatment in postmenopausal women with low bone density: Relations among density, cortical and trabecular microarchitecture, biomechanics, and bone turnover. J. Bone Miner. Res. 2010 Dec;25(12):2558–71.

7. Laib A, Rüegsegger P. Calibration of trabecular bone structure measurements of in vivo three-dimensional peripheral quantitative computed tomography with 28-μm-resolution microcomputed tomography. Bone. 1999 Jan 1;24(1):35–9.

8. Pistoia W, van Rietbergen B, Lochmüller E-M, Lill CA, Eckstein F, Rüegsegger P. Estimation of distal radius failure load with micro-finite element analysis models based on three-dimensional peripheral quantitative computed tomography images. Bone. 2002 Jun 1;30(6):842–8.
